# Inhibition of transcription leads to rewiring of locus-specific chromatin proteomes

**DOI:** 10.1101/gr.256255.119

**Published:** 2020-04

**Authors:** Deepani W. Poramba-Liyanage, Tessy Korthout, Christine E. Cucinotta, Ila van Kruijsbergen, Tibor van Welsem, Dris El Atmioui, Huib Ovaa, Toshio Tsukiyama, Fred van Leeuwen

**Affiliations:** 1 Division of Gene Regulation, Netherlands Cancer Institute, 1066CX Amsterdam, The Netherlands;; 2 Basic Sciences Division, Fred Hutchinson Cancer Research Center, Seattle, Washington 98109, USA;; 3 Leiden Institute for Chemical Immunology, Leiden University Medical Center, 2333ZC Leiden, The Netherlands;; 4 Oncode Institute, Amsterdam University Medical Center, University of Amsterdam, 1105 AZ Amsterdam, The Netherlands;; 5 Department of Medical Biology, Amsterdam University Medical Center, University of Amsterdam, 1105 AZ Amsterdam, The Netherlands

## Abstract

Transcription of a chromatin template involves the concerted interaction of many different proteins and protein complexes. Analyses of specific factors showed that these interactions change during stress and upon developmental switches. However, how the binding of multiple factors at any given locus is coordinated has been technically challenging to investigate. Here we used Epi-Decoder in yeast to systematically decode, at one transcribed locus, the chromatin binding changes of hundreds of proteins in parallel upon perturbation of transcription. By taking advantage of improved Epi-Decoder libraries, we observed broad rewiring of local chromatin proteomes following chemical inhibition of RNA polymerase. Rapid reduction of RNA polymerase II binding was accompanied by reduced binding of many other core transcription proteins and gain of chromatin remodelers. In quiescent cells, where strong transcriptional repression is induced by physiological signals, eviction of the core transcriptional machinery was accompanied by the appearance of quiescent cell–specific repressors and rewiring of the interactions of protein-folding factors and metabolic enzymes. These results show that Epi-Decoder provides a powerful strategy for capturing the temporal binding dynamics of multiple chromatin proteins under varying conditions and cell states. The systematic and comprehensive delineation of dynamic local chromatin proteomes will greatly aid in uncovering protein–protein relationships and protein functions at the chromatin template.

Many proteins assemble on to DNA to implement gene regulatory programs and ensure the expression of a subset of genes in agreement with the state and environmental cues of the cell. RNA polymerase II (Pol II) is an integral part of this assembly as it catalyzes the DNA-dependent synthesis of messenger RNA (mRNA). Regulation of Pol II occurs at different stages of transcription and involves concerted actions of many proteins and protein complexes and dynamic post-translational modifications of histones and the carboxyl-terminal domain (CTD) of Pol II (Sainsbury et al. 2015; Chen et al. 2018; Li et al. 2018).

The process of transcription involves distinct stages: initiation, elongation, and termination (Sainsbury et al. 2015; Chen et al. 2018; Li et al. 2018). The wrapping of DNA by histones into nucleosomes presents a barrier for each of these steps. Chromatin remodelers recruited to promoters can displace nucleosomes to open up crucial recognition elements for transcription factor (TF) binding (Struhl and Segal 2013; Prasad et al. 2016). For initiation of transcription, Pol II assembles with basal TFs (TFIIB-H) at the promoter to form the preinitiation complex, which opens up the DNA, initiates RNA synthesis, and stimulates the escape of Pol II from the promoter (Sainsbury et al. 2015; Feng et al. 2016; Hantsche and Cramer 2017). Productive elongation involves recruitment of Pol II associated factor complex (Paf1C), DRB sensitivity inducing factor (DSIF), Spt4/5, Spt6, and facilitator of chromatin transcription (FACT) (Xu et al. 2017; Ehara and Sekine 2018; Vos et al. 2018). Toward the end of genes, transcription is terminated and the RNA is processed by the recruitment of cleavage and polyadenylation factors, which results in the release of Pol II and the nascent mRNA from the DNA template (Porrua and Libri 2015). Biochemical and genetic studies have provided a rich catalog of factors involved in the different stages of transcription. However, how the many different interactions are coordinated at any given chromatin locus in time and under changing conditions is still poorly understood (Qiu and Kaplan 2019).

Unraveling the relationships between proteins and the hierarchies among them at the chromatin template will require measuring the chromatin interactome and monitoring the changes upon perturbation. A common and convenient strategy to perturb transcription is the use of inhibitors of Pol II (Bensaude 2011). Some of the commonly used inhibitors of transcription initiation or elongation are not active in live yeast cells because of uptake deficiency or because the target protein or target site on the protein is not conserved (Bensaude 2011). However, the addition of the metal chelators thiolutin and 1,10-phenanthroline (PH) leads to rapid loss of transcription in yeast. This has, for example, enabled the determination of mRNA half-lives and establishing the role of Pol II activity in nucleosome positioning (Adams and Gross 1991; Grigull et al. 2004; Sun et al. 2013; Hsieh et al. 2015; Vasseur et al. 2016; Lauinger et al. 2017). In addition, 6-azauracil (AU), which perturbs the supply of ribonucleotides and thereby indirectly affects RNA polymerases, is frequently used to assess transcription elongation phenotypes in yeast (Handschumacher and Welch 1956; Exinger and Lacroute 1992; Shaw and Reines 2000; Mason and Struhl 2005; Zhou et al. 2015). Although these chemicals and related compounds have been used extensively and are convenient and powerful tools to block transcription, little is known about how the direct and indirect inhibition of transcription affects the interactions of Pol II and its protein partners with the chromatin template.

Transcription is also regulated under physiological conditions during development and differentiation. In budding yeast, a global change in transcription is observed when cells enter quiescence, a survival mode that involves a G1-like cell-cycle arrest, with increased resistance to stress, metabolic rewiring, and large-scale reorganization of the genome and cellular machineries (Radonjic et al. 2005; Aragon et al. 2008; Broach 2012; Miles et al. 2013; Kuang et al. 2014; Mews et al. 2014; Guidi et al. 2015; McKnight et al. 2015; Rutledge et al. 2015; Young et al. 2017; Sagot and Laporte 2019a,b; Swygert and Tsukiyama 2019). Quiescence is central to many important biological processes and is conserved from unicellular eukaryotes to multicellular organisms (Cheung and Rando 2013; Sagot and Laporte 2019a). We recently showed that quiescent (Q) cells show a 30-fold drop in mRNA levels and that this massive transcriptional shutoff is dependent on the conserved histone deacetylase (HDAC) Rpd3 (McKnight et al. 2015). Rpd3 is recruited to the majority of gene promoters in Q cells, leading to global hypoacetylation of chromatin and gene repression (McKnight et al. 2015). Further, transcriptional shutdown correlates with loss of Rpb3, one of the subunits of Pol II (McKnight et al. 2015; Young et al. 2017).

To systematically interrogate the chromatin changes at a transcriptionally active locus upon inhibition of transcription, we used Epi-Decoder, a tag–chromatin immunoprecipitation (ChIP)–barcode (BC)-sequencing technology in budding yeast (Korthout et al. 2018). Epi-Decoder enables the decoding of the proteome of a single barcoded genomic locus by DNA sequencing and BC counting. It takes advantage of the power of cellular DNA barcoding (Yan et al. 2008; Fowler and Fields 2014; Vlaming et al. 2016; Chabbert et al. 2018; Kebschull and Zador 2018; Roy et al. 2018) and yeast genetics (Duina et al. 2014) and provides a quantitative approach orthogonal to capture-mass-spectrometry efforts (Schmidtmann et al. 2016; Wierer and Mann 2016; Myers et al. 2018). Here we describe an expanded library of yeast strains carrying a double-BC transcribed reporter gene integrated at the *HO* locus (Epi-Decoder-HO). Of this library, we used a dedicated subset of approximately 700 (putative) chromatin proteins (for more details, see Supplemental Materials and Methods) of which the binding at the reporter locus can be assessed in parallel and in triplicate with three independent BCs in a single sample. This Chrom-3×BC library was used to determine the local chromatin-proteome rewiring at the barcoded transcribed locus in response to transcription inhibition by chemical perturbation and during quiescence.

## Results

### Generation of improved Epi-Decoder-HO libraries

**Figure 1.**
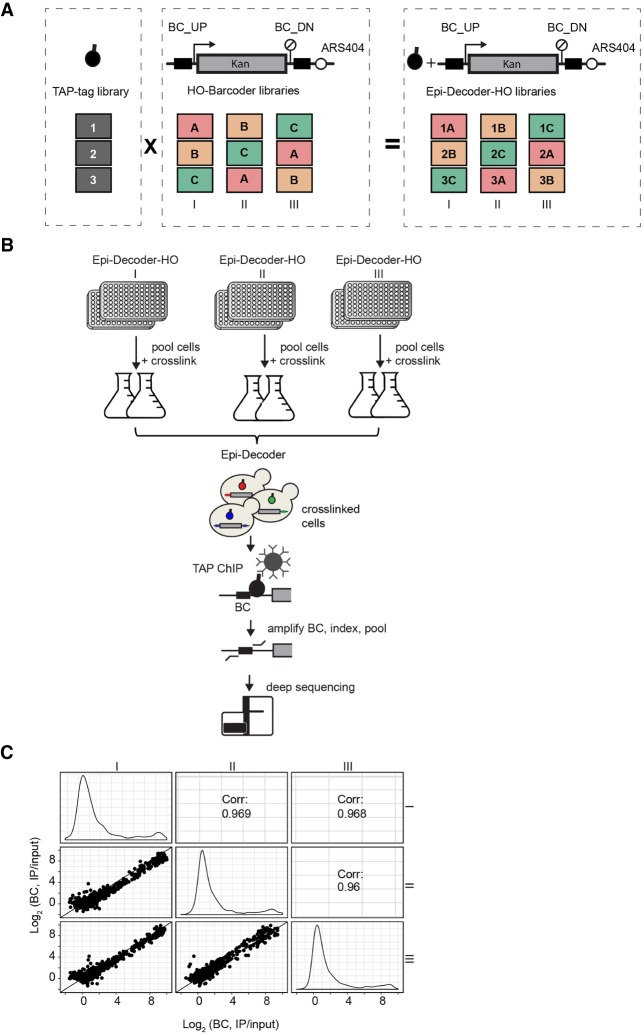
Outline of expanded and optimized Epi-Decoder analysis. (*A*) Three Epi-Decoder-HO libraries were generated by crossing the TAP-tag library to an expanded HO-BC library in three different ways to combine each TAP-tag protein with three independent barcodes (BCs) (see also Supplemental Fig. S1A). The *HO*-BC locus consists of a constitutively expressed 1.5-kb *KanMX* resistance gene integrated at the *HO* locus, controlled by the heterologous *AgTEF1* promoter and terminator from *Ashbya gossypii*, and flanked by a promoter-proximal BC_UP and a terminator-proximal BC_DN. Downstream from BC_DN lies an origin of replication (ARS404). (*B*) Clones of each Epi-Decoder-HO library are combined and processed in two separate pools and used for ChIP of TAP-tagged proteins (spheres with black handle). The BCs (colored lines), which flank the *KanMX* reporter gene (gray box) at the *HO* locus, are amplified from ChIP and input and indexed, allowing for the pools to be combined and counted by massive parallel sequencing. The relative BC count (IP/input) reports on protein abundance of each TAP-tagged protein (approximately 4250) at the barcoded locus. (*C*) Comparison of the binding scores (IP/input) of both BC_UP and BC_DN of chromatin binders (as determined previously by Korthout et al. 2018) in the three Epi-Decoder-HO libraries. Indicated are the Spearman’s correlation coefficients, and the diagonal line represents *x* = *y*. Density plots show the distribution of the BC counts in each of the three replicates. For counts of all proteins examined, see Supplemental Table S1. The results for BC_UP and BC_DN separately are shown in Supplemental Figure S1, B and C.

To measure local proteome dynamics at a promoter and terminator region upon transcriptional inhibition, we first improved the previously reported Epi-Decoder-HO library to optimize the comparison of multiple time points and conditions. Epi-Decoder is a strategy for decoding the local proteome of a single genomic locus (Korthout et al. 2018). It relies on short (∼16- to 20-bp) DNA BCs integrated at a common locus in the genome. Here we used the constitutively expressed *KanMX* marker gene integrated at the *HO* locus and flanked by two BCs—BC_UP (promoter region) and BC_DN (terminator region)—that are 1.5 kb apart (HO-Barcoders). The barcoded *KanMX* cassette is a kanamycin gene controlled by the heterologous *AgTEF1* promoter and terminator from *Ashbya gossypii*, a yeast related to *Saccharomyces cerevisiae*. This 1.5-kb reporter-gene cassette replaces the coding sequence of the *HO* gene and is therefore flanked by the endogenous *HO* promoter and terminator sequences as well as an origin of replication (ARS404) 53 bp downstream from BC_DN. This reporter locus has previously been used in screens for various chromatin- and transcription-related features (Verzijlbergen et al. 2011; Chen et al. 2013; Vlaming et al. 2016, 2019; Korthout et al. 2018). The library of HO-Barcoders was combined with a genome-wide library of proteins tagged with a common epitope tag, a tandem affinity purification (TAP) tag (Fig. 1A; Supplemental Fig. S1A). Upon pooling, cross-linking, ChIP, amplification of the barcoded regions, and counting the BCs by massive parallel sequencing (Fig. 1B), the abundance of each BC (ChIP/input) reports on the occupancy of each tagged protein at its barcoded locus (Verzijlbergen et al. 2011; Vlaming et al. 2016; Korthout et al. 2018). Integral for high-throughput assessments like this is the ability to multiplex many individual TAP-tag clones (approximately 4800 in total) in the ChIP assays using unique BCs and indexing strategies. Here we expanded the previous set of HO-Barcoders from about 1100 to about 2500 (Douglas et al. 2012), enabling coverage of the full Epi-Decoder library with only two separate subsets (Fig. 1B; Supplemental Fig. S1A). The expanded Barcoder-HO library was combined with the TAP-tag library in three different ways (see Methods) such that every TAP-tag is linked to three independent HO-Barcoders (I, II, and III) and that a triplicate TAP-tag subset of approximately 700 chromatin proteins (Chrom-3×BC) was created that can be processed as one pool owing to nonoverlapping BC pairs in the replicates.

### Interrogation of protein binding with multiple DNA BCs

Epi-Decoder uses DNA BC counting as a reporter for protein binding (Korthout et al. 2018). With the three independent Epi-Decoder-HO libraries (Fig. 1B), we first assessed to what extent variation in quantification of protein binding is caused by the BC sequences. The protein-binding patterns in each of the three Epi-Decoder-HO libraries strongly correlated between the replicates (Fig. 1C) for both BC_UP and BC_DN (Supplemental Fig. S1B,C; Supplemental Table S1), and this was further confirmed by the analysis of individual clones (see below). Although it is possible that specific BC sequences could cause a bias for detection of certain proteins, in general our results show that the inferred protein-binding scores are largely independent of the short BC sequences. The use of multiple Tag-Barcode combinations further increases the confidence of single-BC measurements. For example, we have previously shown that Ssl2 and Tfa2, two factors known for their roles in transcription initiation, also show high binding at the *HO* terminator region (Korthout et al. 2018). By replicating this finding with three independent barcodes, we can now negate the possibility that BC effects caused binding of Ssl2 and Tfa2 at the *HO* terminator region (Supplemental Table S1). The consistent and robust binding of Ssl2 and Tfa2 at the terminator region in the absence of other basal TFs suggests a noncanonical function of these factors.

### A chromatin TAP-tag subset library for capturing dynamic local chromatin proteomes

**Figure 2.**
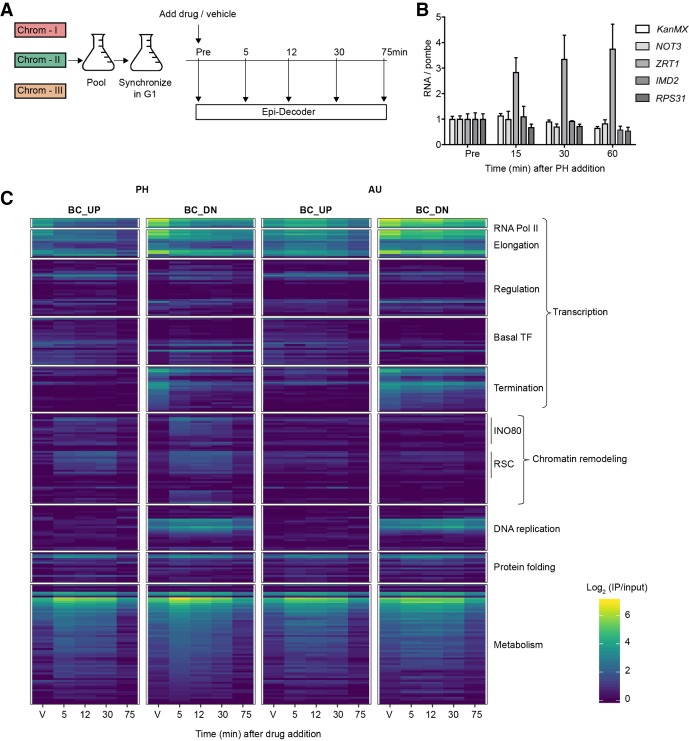
Chromatin-proteome dynamics at the Barcoded-*HO* locus upon chemical inhibition of transcription. (*A*) Three versions of the chromatin TAP-tag subset, each containing a unique set of BC pairs, were combined into one pool (Chrom-3×BC). The culture was incubated with a-factor pheromone to synchronize the cells in G1, after which PH or AU was added and samples were collected at the time points indicated for Epi-Decoder analysis, RNA analysis, and flow cytometry. A sample treated with vehicle was collected at the last time point, and a control sample was taken of a G1-arrested culture without treatment. All experiments were performed at 16°C to facilitate capturing dynamic binding events. (*B*) Analysis of mRNA changes over time by RT-qPCR confirmed the decay of most transcripts and an increase in the PH-responsive *ZRT1* gene under conditions shown in panel *A*. RNA levels are relative to untreated (Pre) and normalized to a transcript from a spike-in of untreated *Schizosaccharomyces pombe* cells (see Methods) to correct for global changes (mean of three biological replicates ± SD). (*C*) Heatmap of the binding scores (mean IP/input of three biological replicates) of selected proteins with a binding score >0.5 at any of the four local proteome time series indicated. For mean binding scores of all proteins examined in the Chrom-3×BC library, see Supplemental Table S2. Proteins were manually clustered and ranked in functional subcategories.

Having confirmed that Epi-Decoder provides a robust assay to quantitatively measure protein binding at a single locus of thousands of proteins in parallel, we focused on the chromatin TAP-tag subset (Chrom-3×BC; see Methods) (Fig. 2A) to determine the dynamics of the local chromatin proteome following chemical inhibition of Pol II. The antifungal agent PH is a metal ion chelator that sequesters Zn^2+^ ions, which are essential for the activity of RNA polymerases (Scrutton et al. 1971; Markov et al. 1999; Cramer et al. 2000; McCann et al. 2012). The addition of PH to cells rapidly affects mRNA levels (Grigull et al. 2004; Miller et al. 2011; Wada and Becskei 2017). AU is an inhibitor of IMP dehydrogenase (IMPDH), the rate-limiting enzyme in de novo GTP synthesis (Grigull et al. 2004; Kaplan et al. 2012; Ljungdahl and Daignan-Fornier 2012). The treatment of cells with AU results in depletion of intracellular nucleotide pools, thereby affecting transcription elongation (Handschumacher and Welch 1956; Exinger and Lacroute 1992; Shaw and Reines 2000; Shaw et al. 2001; Mason and Struhl 2005). To investigate the consequences of inhibition of Pol II at the chromatin template, the Chrom-3×BC Epi-Decoder library was incubated with PH and AU, and the local proteomes were determined at several subsequent time points (Fig. 2A). All experiments were performed at low temperature (16°C) to facilitate the capturing of dynamic binding events. In addition, cells were arrested in G1 to avoid cell-cycle-dependent effects (Supplemental Fig. S2A; O'Duibhir et al. 2014). Independent triplicate measurements in the Chrom-3×BC pool and sample multiplexing enabled efficient analysis of the dynamics of known chromatin proteins across multiple conditions and time points.

We confirmed that PH and AU are active under these conditions: Both compounds inhibited cell growth (Supplemental Fig. S2B), and addition of PH led to induction of *ZRT1* mRNA whereas addition of AU led to induction of *IMD2* mRNA (Fig. 2B; Supplemental Fig. S2C), which is in agreement with previous observations (Shaw and Reines 2000; Grigull et al. 2004) that inhibition of transcription by these drugs is not complete. Under these experimental conditions and time points, we observed little decay of mRNA levels of the barcoded *KanMX* gene or other genes examined (Fig. 2B; Supplemental Fig. S2C), in agreement with the observed temperature effects on RNA decay kinetics (Lotan et al. 2005). In contrast, global inspection of binders at the barcoded *KanMX* gene showed that the addition of PH or AU led to many rapid and pronounced changes at the chromatin level at BC_UP and BC_DN (Fig. 2C). Generally, changes in protein binding were stronger in PH than in AU, confirming that the observed effects are specific for the treatment.

### Rewiring of the core transcription machinery at chromatin upon inhibition of Pol II

**Figure 3.**
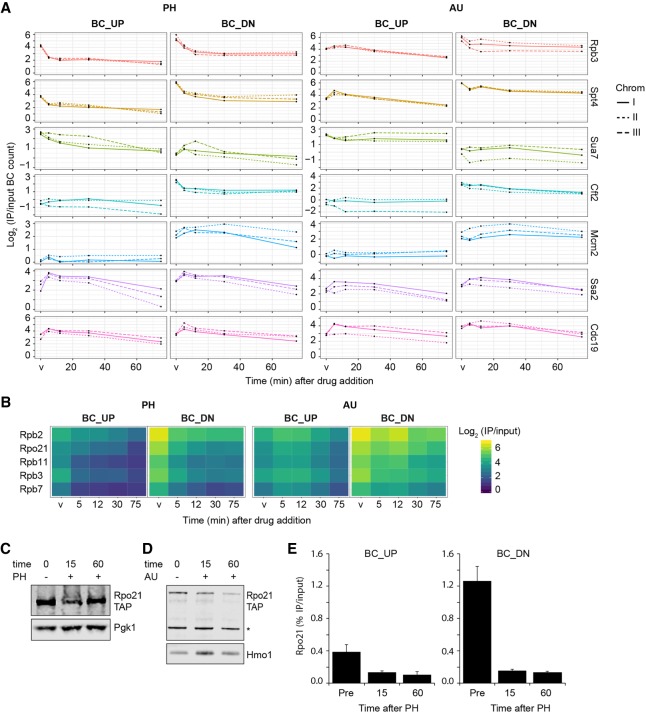
Treatment with phenanthroline (PH) leads to rapid loss of Pol II and transcription-associated proteins. (*A*) Differential and dynamic binding behavior of proteins representing different chromatin processes upon treatment with PH and AU. The lines indicate the three different BC pairs of the indicated TAP-tagged proteins in the Chrom-3×BC library (Log_2_ IP/input at time points indicated in Fig. 2). (*B*) Zoom-in of heatmap of Figure 2 showing the Pol II subunits present in the library. (*C*,*D*) Immunoblot analysis of the largest subunit of Pol II (Rpo21-TAP) with and without PH and AU treatment in G1-arrested cells at 16°C. Pgk1, Hmo1, and a nonspecific band (*) were used as loading controls. (*E*) ChIP-qPCR analysis of Rpo21 binding at the BC_UP and BC_DN regions in G1-arrested cells treated with (15 and 60 min) and without (Pre) PH at 16°C (average of three biological replicates ± SD).

The global overview of the dynamic chromatin proteomes (average of three replicates) (Fig. 2C) revealed broad rearrangements, especially of protein complexes related to transcription. Other proteins were unaffected, such as the replication proteins specifically associated with BC_DN proximal to the origin of replication ARS404. This was further illustrated by the analysis of individual replicate measurements of proteins representing different aspects of chromatin biology (Fig. 3A). Because these three replicates were based on different BC pairs, the reproducible results indicate that BC sequences did not generally affect the protein-binding measurements in Epi-Decoder. As observed previously (Korthout et al. 2018), Pol II and transcription elongation factors were found at BC_UP and were more abundant at BC_DN, initiation factors and basal TFs were more enriched at BC_UP, and transcription termination factors and replication proteins were specifically bound to BC_DN (Figs. 2C, 3A; Supplemental Table S2). To determine the consequences of transcription inhibition for the transcription machinery in more detail, we focused on Pol II and transcription elongation factors. All the Pol II subunits present in our Epi-Decoder library showed a pronounced and progressive loss of binding upon addition of PH at BC_UP and BC-DN. In AU, Pol II binding was also reduced but to a lesser extent (Fig. 3B). Therefore, we here focused on the changes following treatment with PH. Loss of binding can be caused by lower levels of the protein owing to protein degradation or by redistribution of the protein. To distinguish between these two possibilities, we determined the protein level of Rpo21, the largest subunit of Pol II, by immunoblot analysis and found that treatment with PH did not lead to reduced Rpo21 protein levels (Fig. 3C; Supplemental Fig. S3A), whereas treatment with AU showed a modest decrease (Fig. 3D; Supplemental Fig. S3B). Therefore, the reduced binding of Pol II subunits in PH was not accompanied by increased Pol II protein degradation.

Finally, the strong reduction in Pol II binding observed in Epi-Decoder in PH at the barcoded *KanMX* gene under the control of the *AgTEF1* promoter and terminator could be validated by ChIP-qPCR at the 5′ and 3′ ends of the endogenous *TEF1* gene (Fig. 3E; Supplemental Fig S3C). Thus, in addition to the current knowledge that Pol II activity is inhibited directly by PH and indirectly by AU (Scrutton et al. 1971; Markov et al. 1999; Cramer et al. 2000; McCann et al. 2012), our results show that chemical inhibition of Pol II also leads to partial eviction of the core Pol II machinery from the chromatin template.

### Epi-Decoder uncovers protein–protein relationships

**Figure 4.**
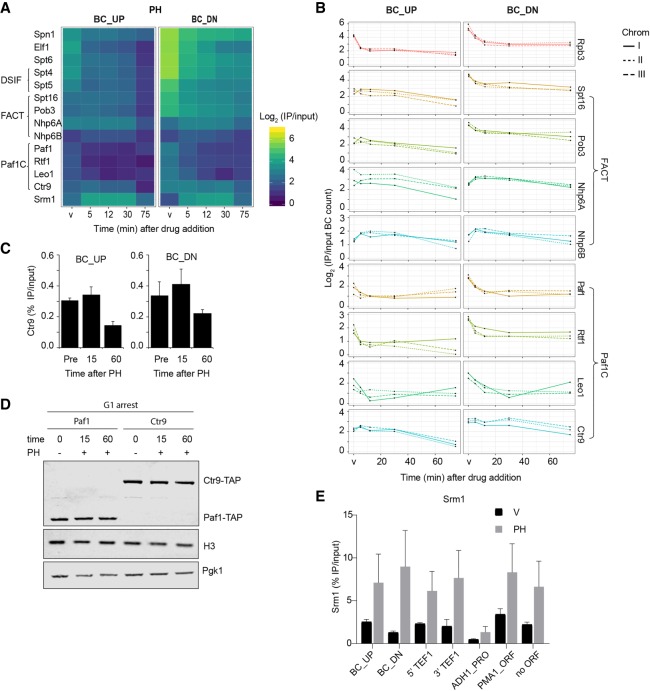
Differential response of transcription elongation factors to chemical inhibition of Pol II. (*A*) Zoom-in on the heatmap of Figure 2 (PH treatment), showing proteins annotated to transcription elongation. (*B*) Independent replicates of proteins related to FACT and Paf1C (Log_2_ IP/input at time points as in Fig. 2). The lines show the three different BC pairs of the indicated TAP-tagged proteins in the Chrom-3×BC library. (*C*) ChIP-qPCR analysis of Ctr9 binding at the BC_UP and BC_DN regions in G1-arrested cells treated with (15 and 60 min) and without (Pre) PH at 16°C (average of three biological replicates ± SD). (*D*) Immunoblot analysis of Ctr9-TAP and Paf1-TAP with and without PH treatment in G1-arrested cells at 16°C. Pgk1 and H3 were used as loading controls. (*E*) ChIP-qPCR analysis of Srm1 binding in G1-arrested cells at 16°C, treated for 15 min with vehicle (V) or PH (average of three biological replicates ± SD). Analyzed loci are the BC_UP and BC_DN regions, the 5′ and 3′ end of the endogenous *TEF1* gene, the *ADH1* promoter, the *PMA1* open reading frame, and a nontranscribed locus (for more details, see Supplemental Table S5; van Welsem et al. 2018).

Several proteins and protein complexes have been biochemically or genetically linked to the process of transcription elongation (Koerber et al. 2009; Venters and Pugh 2009; Kwak and Lis 2013; Pufall and Kaplan 2013; Cucinotta and Arndt 2016; Van Oss et al. 2017; Chen et al. 2018; Vinayachandran et al. 2018). However, how the interactions between the core transcriptional machinery and elongation factors are dynamically orchestrated on the chromatin template is still incompletely understood (Mayer et al. 2010; Vinayachandran et al. 2018). Inspection of the dynamic proteomes at the *HO* locus showed that elongation factors did not all respond equally to inhibition of Pol II by PH (Fig. 4A) and AU (Supplemental Fig. S4A). The conserved elongation factors Elf1, Spn1, Spt6, and DSIF (Spt4/5) were rapidly and progressively evicted from chromatin, closely resembling the dynamics of Pol II upon PH treatment (Figs. 3B, 4A). This indicates that most elongation factors depend on Pol II for recruitment and maintenance. The treatment with AU, performed and processed in parallel, showed more modest effects on binding of Pol II and the elongation factors (Supplemental Fig. S4A,B).

The FACT complex, composed of Spt16 and Pob3, is known to be recruited to chromatin by the act of transcription and its associating factors Nhp6a and Nhp6a, two high mobility group (HMG) proteins (Ruone et al. 2003; Kwak and Lis 2013; McCullough et al. 2015; Martin et al. 2018; Pathak et al. 2018). At BC_UP, FACT eviction was delayed compared with eviction of Pol II upon PH treatment (Fig. 4A,B), suggesting that FACT binding might depend on more stable factors at transcribed genes such as histones or histone modifications (Martin et al. 2018; Vinayachandran et al. 2018). At BC_DN, the two core FACT subunits Spt16 and Pob3 were rapidly lost upon addition of PH, closely resembling the behavior of Pol II and other elongation factors. In contrast, the HMG proteins Nhp6a and Nhp6b did not show reduced binding after Pol II inhibition (Fig. 4A,B). This difference may reflect the ability of HMG proteins to bind DNA and nucleosomes directly, independently of other chromatin proteins.

Recent structural studies show that Paf1C, DSIF (Spt4/5), and Spt6 can form an intricate protein network around elongation-permissive Pol II (Vos et al. 2018; Xie et al. 2018). Indeed, like DSIF and Spt6, most Paf1C subunits showed reduced binding to chromatin when Pol II was inhibited and evicted by PH and AU (Fig. 4A,B; Supplemental Fig S4A,B). However, binding of Ctr9, the largest subunit and a key scaffold protein of Paf1C (Deng et al. 2018; Vos et al. 2018; Xie et al. 2018), was largely unaffected in PH, with a small decrease only observed at later time points (Fig. 4A,B). We confirmed the delayed loss of Ctr9 by ChIP-qPCR analysis of the barcoded promoter and terminator regions (Fig. 4C), as well as the endogenous *TEF1* locus (Supplemental Fig. S4C). The differences in kinetics were not caused by differences in protein abundance, because Ctr9 showed very similar global expression levels as the Paf1 and Pol II subunits (Supplemental Fig. S4D). We also tested whether the delayed loss of Ctr9 was caused by a difference in crosslinking sensitivity (Zaidi et al. 2017; De Jonge et al. 2019). However, Paf1 and Rpo21 were not more sensitive to shorter cross-linking conditions than was Ctr9 (Supplemental Fig. S4E). Finally, addition of PH did not lead to major changes in the expression level of Rpo21 and Paf1, compared with Ctr9, the latter remaining bound when Rpo21 and Paf1 had already been evicted upon PH treatment (Fig. 4D). Together, our results show that binding of Ctr9 at transcribed chromatin is independent of Pol II and Paf1C subunits, suggesting that Ctr9 has a transcription elongation–independent mechanism to bind to DNA, perhaps mediated by its known interactions with DNA and nucleosomes (Musso et al. 2000; Deng et al. 2018; Vos et al. 2018). These binding activities may be responsible for anchoring Ctr9 to PH-perturbed chromatin in the absence of its complex partners and Pol II. Of note, under conditions of transcriptional repression in Q cells, Ctr9 followed the pattern of the other Paf1C members (see below), showing that its dynamics are context dependent.

In addition to uncovering protein-binding dynamics within well-known protein complexes, the parallel analysis of hundreds of proteins also offers the possibility to obtain more insight into proteins of which the chromatin functions have been less well annotated. Here we more closely examined Srm1 (also known as Prp20), a homolog of human Regulator of Chromosome Condensation 1 (RCC1). Srm1/RCC1 is a guanine nucleotide exchange factor that localizes to the nucleus, binds nucleosomes in a dynamic way, and occupies a large part of the yeast genome (Nemergut et al. 2001; Koerber et al. 2009; Makde et al. 2010; Wu et al. 2011; Bierbaum and Bastiaens 2013; McGinty and Tan 2016). In the Epi-Decoder analysis, all proteins are examined in one pool and with the same tag, thereby delivering qualitative as well as relative quantitative information. The high BC counts observed for Srm1 show that it binds efficiently to chromatin, in the same range as the abundant transcription elongation factors mentioned above (Fig. 4A; Supplemental Fig. S4A). Analysis of Srm1 by ChIP-qPCR confirmed the efficient binding of this protein and an increase after PH treatment at the reporter locus as well as endogenous loci, including a nontranscribed region (Fig. 4E). This, together with the genome-wide binding pattern (Koerber et al. 2009), indicates that Srm1 should be considered as a common component of yeast chromosomes. However, the distribution and kinetic behavior of Srm1 did not mirror that of the transcription elongation factors: Upon treatment with PH, Srm1 binding increased (Fig. 4A,E), whereas binding of Pol II and most elongation proteins decreased. Although not quantitatively the same, the behavior of Srm1 is more similar to that of the chromatin remodelers INO80 and RSC, several metabolic enzymes (Fig. 2C), the heat shock protein Ssa2 (Fig. 3A; Supplemental Fig. S4F), and HMG protein Hmo1 (Supplemental Fig. S4F). In contrast, the HMG-like protein Spt2, a chaperone involved in histone recycling over transcribed regions of active genes (Nourani et al. 2006; Chen et al. 2015), was decreased in active regions (Supplemental Fig. S4F). The dynamic behavior of Srm1, Ssa2, Hmo1, and Spt2 after PH treatment was not caused by changes in global protein levels (Supplemental Fig. S4G). Together, these results suggest that Srm1 is an abundant chromatin protein with the potential to affect chromatin structure and function but that it might not act as a canonical transcription elongation factor.

### Rearrangement of the chromatin proteome of the barcoded *HO* locus upon entry in quiescence

Finally, we investigated how the chromatin proteome of the barcoded *HO* locus was altered by the strong physiological transcriptional shut down during quiescence. Chrom-3×BC Epi-Decoder library pools were grown in YEPD for 7 d at 30°C , which causes cells to arrest in saturation with G1 DNA content. Stationary phase cultures consist of two populations: Q cells, which are more uniform, long-lived, and stress resistant, and nonquiescent (NQ) cells, which are more heterogeneous, short-lived, and stress sensitive (Allen et al. 2006; Aragon et al. 2008; Li et al. 2013; Young et al. 2017; Sagot and Laporte 2019a,b). NQ cells were also isolated and processed as a reference.

**Figure 5.**
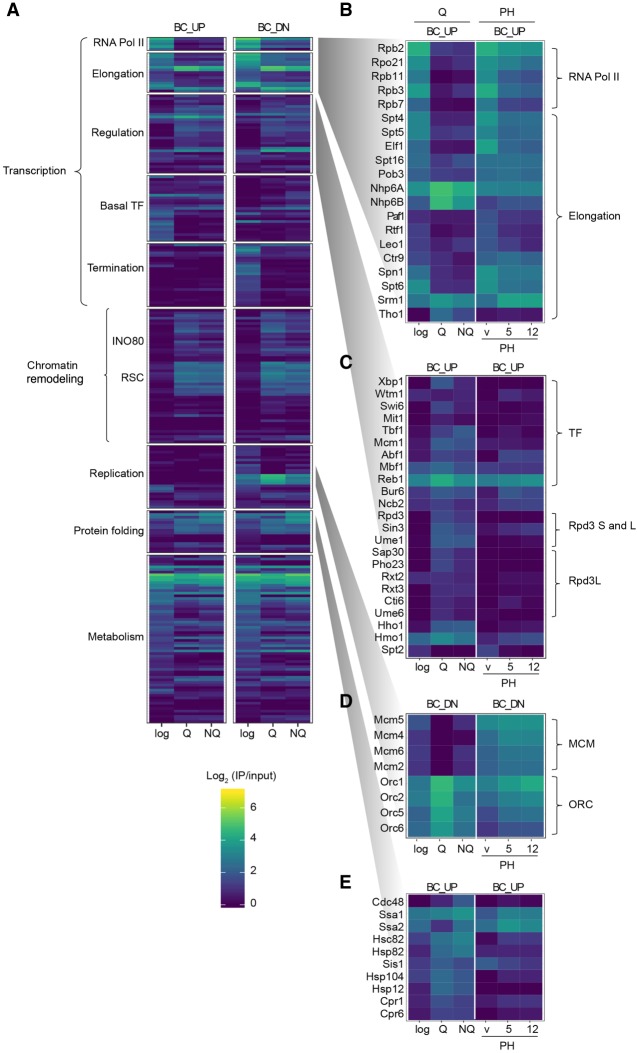
Chromatin-proteome rewiring upon transcriptional repression in quiescence. (*A*) Heatmap of the HO Epi-Decoder binding scores in mid-log, Q, and NQ cells (mean IP/input of three biological replicates). For mean binding scores of all proteins examined in the Chrom-3×BC library, see Supplemental Table S3. Proteins were manually clustered and ranked in functional subcategories as in Figure 2C. (*B*–*E*) Zoom-in of heatmap of panel *A* (promoter region BC_UP, except replication for which terminator BC_DN next to the origin of replication was used) showing proteins in the indicated annotated clusters. PH treatment during G1 arrest (vehicle, 5 and 12 min) is shown for comparison.

Analysis of the RNA Pol II machinery, transcription elongation factors, basal TFs, and transcription termination and RNA processing factors showed a pronounced loss of the core transcription machinery in Q and NQ cells (Fig. 5A,B; Supplemental Fig. S5A–E; Supplemental Table S2). Overall, the magnitude of the changes was higher than that observed in the G1 cells treated with PH with several exceptions. The FACT-associating factors Nhp6a and Nhp6a showed increased chromatin binding in Q cells (Fig. 5B; Supplemental Fig. S5A), in contrast to PH-treated cells (Fig. 4; Supplemental Fig. S4). Tho1, a protein associated with transcribed chromatin, showed increased abundance in Q and NQ cells but not in PH (Fig. 5B; Supplemental Fig. S5A), suggesting that Tho1 might have Q/NQ cell–specific functions and that its recruitment is independent of ongoing transcription, in contrast to canonical transcription elongation proteins.

To gain more insight into the transcriptional repression of the barcoded *HO* locus, we inspected transcriptional regulators at the BC_UP promoter region (Fig. 5C; Supplemental Fig. S5B). Q cells showed binding of the quiescence-specific transcription repressor Xbp1, which was absent from mid-log cells and during PH treatment. This protein is known to recruit the HDAC Rpd3, mediating the global shutdown of gene expression in Q cells (McKnight et al. 2015). Indeed, Rpd3 and members of the Rpd3L complex were mostly undetectable in mid-log but readily detected in Q cells. Members unique for the Rpd3S complex were not detected (Supplemental Table S3). This well-established repression pathway was accompanied by increased abundance of several TFs such as Mcm1, Mbf1, Tbf1, Abf1, and Swi6 (Fig. 5C; Supplemental Fig. S5B).

Besides the core transcriptional machinery, TFs, and Rpd3L, we observed several other changes in the chromatin proteomes (Fig. 5C–E; Supplemental Fig. S5B–G). Yeast Histone H1 (Hho1) showed increased abundance in Q and NQ cells (Fig. 5C; Supplemental Fig. S5B). HMG protein 1 (Hmo1), which has been shown to function as a linker histone and promote chromatin compaction (Panday and Grove 2016), showed the same trend (Fig. 5C; Supplemental Fig. S5B). In contrast, the negative regulator of transcription Spt2 left the chromatin in Q and NQ cells, as it did in PH-treated cells. These changes were paralleled by the recruitment of the ATP-dependent chromatin remodeling complexes INO80 and RSC, as well as several heat shock proteins and other factors involved in protein folding (Fig. 5E; Supplemental Fig. S5C,F). In addition, changes were seen in the binding of metabolic enzymes (Supplemental Fig. S5G). Future functional studies will be required to determine the role of these dynamic interactions in the global transcriptional repression occurring in Q cells.

Finally, quiescence also affected the organization of replication proteins at the origin of replication (ARS404) proximal to BC_DN (Fig. 5D). In contrast to PH treatment, cells in quiescence showed a complete loss of the minichromosome maintenance complex (MCM), the replicative helicase. Q cells are arrested in a G1–like state, but binding of MCM was maintained in cells arrested in G1 by mating pheromone (Fig. 5D; Liang and Stillman 1997). The loss of MCM was accompanied by increased abundance of the members of the origin-recognition complex (ORC) (Fig. 5D). This altered balance is consistent with a competition model that has been proposed for the MCM and ORC (Aparicio et al. 1997). Replication origin licensing, which involves the binding of ORC and subsequently the assembly of an inactive form of the replicative helicase MCM, occurs during late mitosis and the G1 phase of the cell cycle. After formation of a replication complex, during S phase the helicase is activated and the replication initiates (Siddiqui et al. 2013; Bell and Labib 2016). It is possible that clearing MCM from origins in Q cells is important for eliminating any inappropriate activation of replication origins during the prolonged state of arrest. The mechanism causing the absence of MCM and the role thereof in Q-cell biology will require further study, but it should be noted that similar dynamics have been observed in the mouse and *Schizosaccharomyces pombe* (Madine et al. 2000; Sun et al. 2000; Namdar and Kearsey 2006). Our results suggest that replication origin licensing must be re-established when cells re-enter the cell cycle.

## Discussion

Determining the changes to chromatin composition in response to cell signaling or stress can reveal insights into common mechanisms of gene regulation. Obtaining a comprehensive understanding of chromatin changes, although highly informative (Kim et al. 2010; Weiner et al. 2012; Vinayachandran et al. 2018), is often laborious and therefore not applicable to multiple conditions. Epi-Decoder provides a strategy for identifying and quantifying in an unbiased and systematic manner the proteome of an individual genomic locus by DNA sequencing. Here we used a dedicated comprehensive chromatin library (Chrom-3×BC) to determine the changes in local chromatin proteomes upon inhibition of Pol II by chemical means or by physiological signals during quiescence.

PH is a potent inhibitor of Pol II and has fungistatic activity against a broad range of pathogenic fungi and bacteria (McCann et al. 2012). PH and thiolutin, which acts in a similar way, are often used to determine the consequences of perturbation of transcription in yeast. However, the consequences at the chromatin level have not been well characterized even though RNA processing, RNA export, and other downstream processes are linked to transcription and are influenced by chromatin modifying factors. Inhibition of Pol II with PH resulted in rapid remodeling of the local chromatin proteomes at the *HO* locus (Fig. 2C). Pol II was evicted, probably reflecting a structural rearrangement caused by PH. This idea is in agreement with observations that mutants of Pol II with altered catalytic activity show reduced Pol II occupancy at genes (Malik et al. 2017). We did not observe changes in overall abundance of the evicted proteins Rpo21 and Paf1 and several other proteins, indicating that the factors evicted in PH were not subject to degradation. Besides inhibiting Pol II, PH—a metal chelator—is known to also affect the activity of metalloproteases, including a proteasome-associated deubiquitinating enzyme (Verma et al. 2002; McCann et al. 2012). Therefore, the stability of the proteins evicted by PH may at least in part be explained by inhibition of proteasome-mediated degradation. The nature and scale of the changes observed after PH treatment, particularly the loss of the core transcription machinery, suggests that inhibition of Pol II can have widespread effects on many nuclear processes in the cell. This should be considered when interpreting downstream effects of chemical inhibitors of transcription, such as mRNA processing and stability.

During quiescence, global changes in histone modifications have been observed (Mews et al. 2014; Young et al. 2017), as well as global repression by Rpd3 (McKnight et al. 2015), chromatin compaction by condensin (Swygert et al. 2019), and reduced Pol II binding (Young et al. 2017). However, the extent to which the epigenome of Q cells is remodeled to support this important developmental state and to allow re-entry into vegetative growth remains poorly understood (Swygert and Tsukiyama 2019). Application of Epi-Decoder to the barcoded *HO* locus revealed that upon loss of transcription during quiescence, the chromatin proteome of a transcribed region undergoes a major rewiring that affects transcriptional proteins and replication complexes. In addition, we observed the recruitment of structural proteins, chromatin remodelers, and protein-folding machineries, as well as changes in metabolic enzymes. Although many changes between mid-log and Q cells were also observed upon PH treatment, we observed Q-cell-specific changes as well, including increased occupancy of other transcription-related proteins (Fig. 5C), Hho1, changes in specific metabolic enzymes (e.g., Tdh1, Arg1; Supplemental Fig. S5E), and differential binding of several heat shock proteins (e.g., Ssa1 versus its paralog Ssa2) (Fig. 5E; Supplemental Fig. S5E). We note that some of the observed dynamics in Q and NQ cells might, at least in part, be driven by altered expression of the proteins in the different cell states. Determining the functional roles of these Q-cell-specific and NQ-cell-specific changes will require further mechanistic studies.

Although Q cells and NQ cells showed overlap in the changes compared with mid-log cells, we also observed important differences, in agreement with the different fates of the long-lived Q and short-lived NQ cells (Li et al. 2013) and the differences in global histone modifications (Young et al. 2017). NQ cells lacked efficient recruitment of Xbp1, which correlated with lower levels of Rpd3L and INO80. In addition, the loss of the MCM complex was incomplete in NQ cells. Together these changes may contribute to the poor fitness of these cells. Furthermore, the binding of Cdc48 at the BC_UP promoter region and BC_DN terminator region in NQ cells is a possible indicator of the general short-lived nature of NQ cells (Fig. 5E). Cdc48, known as VCP in humans, is an essential and conserved AAA+ ATPase that functions as an unfoldase or segregase, facilitating the extraction of proteins from macromolecular complexes, including chromatin, to enable subsequent degradation by the proteasome (Dantuma et al. 2014; Franz et al. 2016). The recruitment of protein quality-control factors to the chromatin agrees with recent observations that effective and diverse protein quality-control mechanisms are active in the nucleus (Jones and Gardner 2016; Prasad et al. 2018; Frottin et al. 2019; Jones et al. 2019).

Our results show that Epi-Decoder provides a powerful strategy for capturing the temporal binding dynamics of chromatin proteins under varying conditions. We expect that future studies on local chromatin-proteome maps under different conditions and at other genomic loci will offer powerful resources for detailed molecular and functional annotation of chromatin proteins and their interactions and relationships at the genome.

## Methods

### Yeast strains and libraries

Yeast strains used in this study are listed in Supplemental Table S4. Library manipulations on solid media were performed using synthetic genetic array (SGA) technology (Tong and Boone 2006) and a ROTOR instrument (Singer Instruments). Yeast media were prepared as previously described (Tong and Boone 2006; Korthout et al. 2018). Details of library construction and growth conditions are provided in the Supplemental Materials and Methods.

### RNA isolation and reverse transcription

RNA was isolated using the RNeasy mini kit (Qiagen) using the protocol for yeast cells, with a few modifications, essentially as previously described (Korthout et al. 2018) and with a spike-in reference (A8545; a gift from R. Allshire) as described in the Supplemental Materials and Methods. RT-qPCR was performed with the primers described in Supplemental Table S5. Each sample was measured in two technical duplicates, and the average value was taken when combining biological replicates.

### Epi-Decoder

Epi-Decoder was performed and analyzed essentially as described previously (Korthout et al. 2018). Protein binding at BC_UP and BC_DN was analyzed separately with specific primers (Supplemental Table S5). Libraries of the PCR products were mixed in an equimolar fashion and sequenced (single read, >50 bp) on a HiSeq 2500/MiSeq platform (Illumina), using one or a mix of custom sequencing primers (Supplemental Table S5). Details of the Epi-Decoder protocol and analysis and the BC counting can be found in the Supplemental Materials and Methods.

### ChIP-qPCR

ChIP-qPCR experiments were performed with IgG Sepharose 6 fast flow beads or epoxy-activated Dynabeads as described previously (Korthout et al. 2018; Vlaming et al. 2019) and in the Supplemental Materials and Methods. Each ChIP was performed in triplicate.

### Protein detection by immunoblot and antibodies

For immunoblotting, strains were grown to mid-log phase or arrested in G1 and processed as described previously (Korthout et al. 2018; Vlaming et al. 2019). Antibodies used and detailed protocols can be found in the Supplemental Materials and Methods.

## Data access

All raw sequencing data generated in this study and corresponding reference tables (Supplemental Tables S6–S11) have been submitted to the NCBI BioProject database (https://www.ncbi.nlm.nih.gov/bioproject/) under accession number PRJNA610036, and to the NCBI BioSample database (https://www.ncbi.nlm.nih.gov/biosample/) under accession numbers SAMN14271070, SAMN14271071, and SAMN14271072. All processed data are within the paper and the Supplemental Material.

## Competing interest statement

The authors declare no competing interests.

## Supplementary Material

Supplemental Material
