# Supplementary material for: Inhibition of transcription leads to rewiring of locus-specific chromatin proteomes
Source: Genome Res. 2020 Apr;30(4):635–46. doi: 10.1101/gr.256255.119 (PMC7197482; doi:10.1101/gr.256255.119)
Supplement: Supplemental Material [file supp_30_4_635__index.html]

Inhibition of transcription leads to rewiring of locus-specific chromatin proteomes — Inhibition of transcription leads to rewiring of locus-specific chromatin proteomes — Supplemental Material 

# Inhibition of transcription leads to rewiring of locus-specific chromatin proteomes

## Supplemental Material

- Supplemental\_Material.pdf
- Supplemental\_Table\_S1.xlsx
- Supplemental\_Table\_S2.xlsx
- Supplemental\_Table\_S3.xlsx
- Supplemental\_Table\_S4.xlsx
- Supplemental\_Table\_S5.xlsx
- Supplemental\_Table\_S6.xlsx
- Supplemental\_Table\_S7.xlsx
- Supplemental\_Table\_S8.xlsx
- Supplemental\_Table\_S9.xlsx
- Supplemental\_Table\_S10.xlsx
- Supplemental\_Table\_S11.xlsx
