# Supplementary material for: Inhibition of transcription leads to rewiring of locus-specific chromatin proteomes
Source: Genome Res. 2020 Apr;30(4):635–46. doi: 10.1101/gr.256255.119 (PMC7197482; doi:10.1101/gr.256255.119)
Supplement: Supplemental Material [file supp_gr.256255.119_Supplemental_Material.pdf]

Supplemental Material

Poramba-Liyanage et al, Inhibition of transcription leads to rewiring of locus-specific chromatin proteomes.

Supplemental Figures

- Supplemental Figure S1
- Supplemental Figure S2
- Supplemental Figure S3
- Supplemental Figure S4
- Supplemental Figure S5

Supplemental Materials and Methods

Supplemental References

Supplemental Figure S1

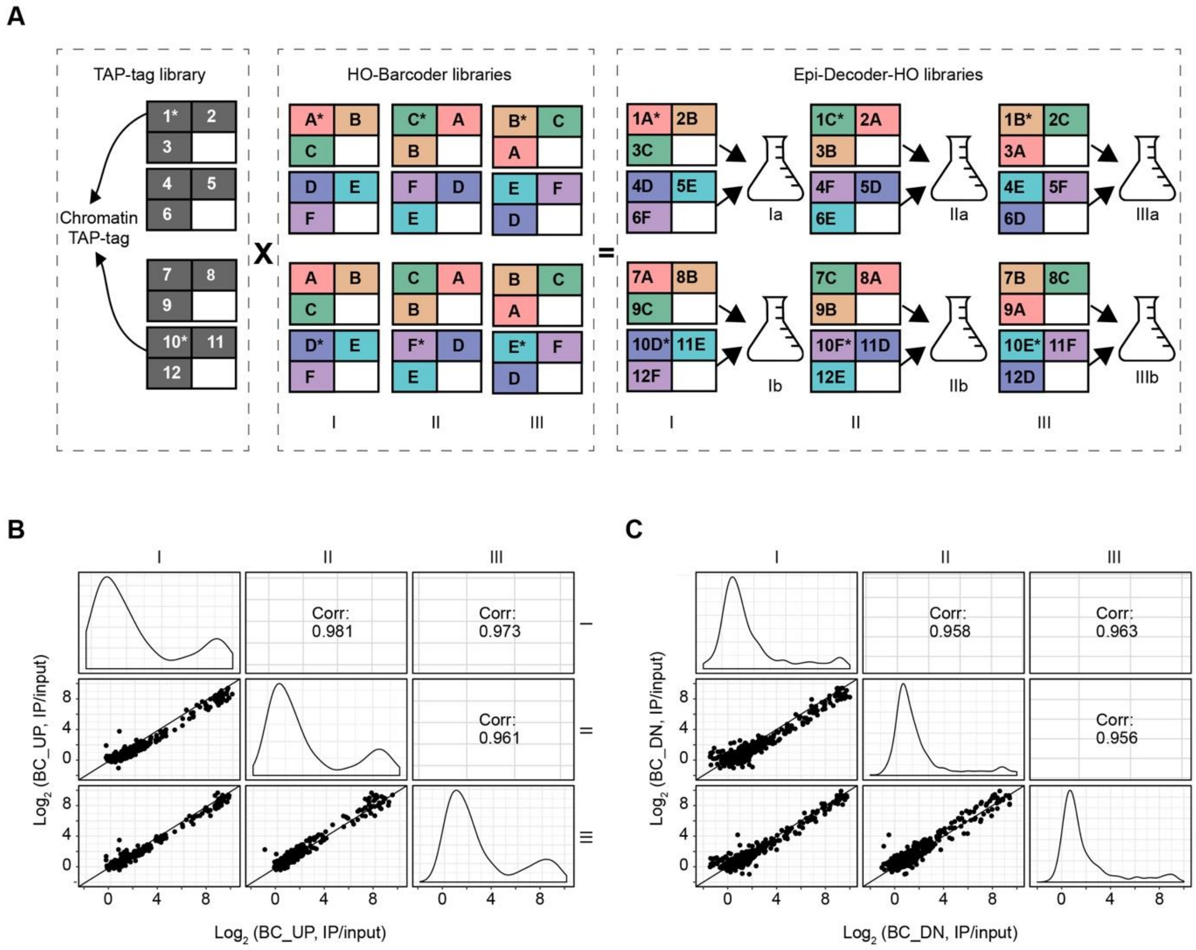

Supplemental Figure S1
(A) *Details of the construction of triplicate Epi-Decoder-HO libraries. A re-arrayed MATα TAP-tag library and an expanded MATa BC library were crossed in three different combinations. In the re-arrayed TAP-tag library, a subset of chromatin proteins was allocated to two 384-well plates (*). The HO-BC library consists of ~2250 BCs. For each cross, the HO-BC library was re-shuffled to obtain three Epi-Decoder-HO libraries, I, II and III with different TAP-tag-BC combinations. Each library was processed in two pools, 'a' and 'b'. The three versions of the Chromatin-TAP-tag subset (*; Chrom-3xBC) have non-overlapping barcodes and were processed in one pool.* ***(B-C)*** *Comparison of the binding scores (IP/input) of BC_UP and BC_DN separately for chromatin binders (as determined previously in (Korthout et al. 2018)) in the three full Epi-Decoder-HO libraries. Indicated are the Spearman correlation coefficients and the diagonal line represent x=y. Density plots show the distribution of the barcode counts in each of the three replicates. See Table S1 for binding scores of all proteins examined.*

Supplemental Figure S2

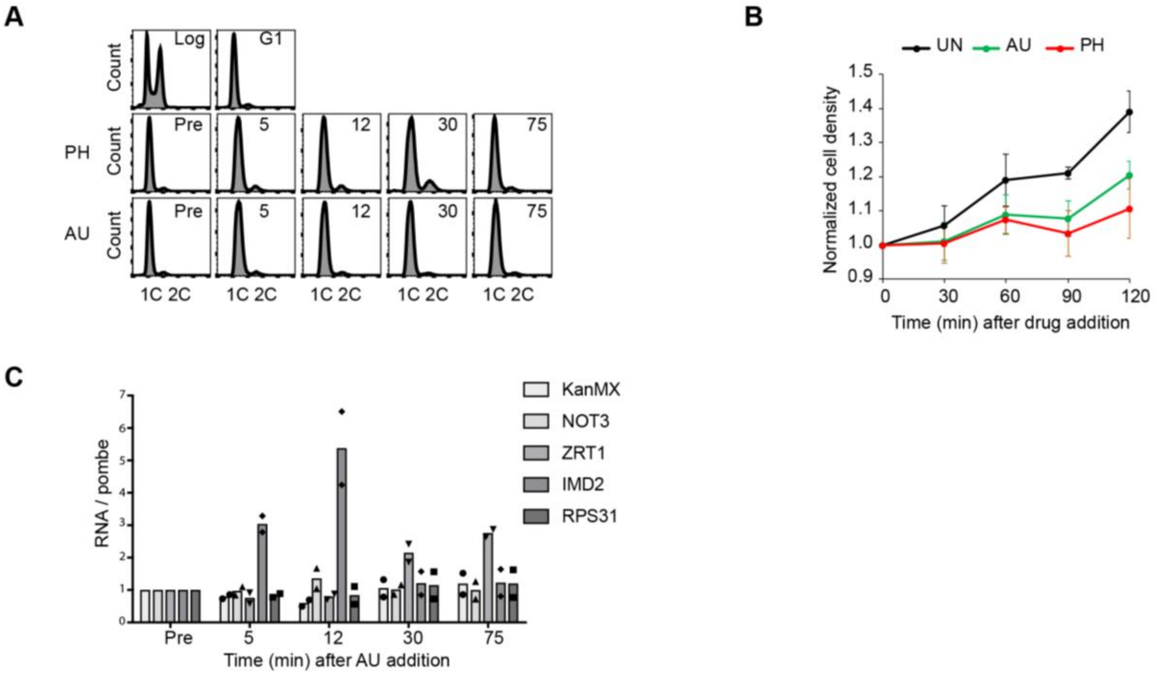

Supplemental Figure S2

(A) *Flow cytometry analysis of DNA content to verify the G1 arrest by a- factor during the course of the experiment show in Fig. 2A. Samples were collected before treatment (Pre) and at the post-treatment time points indicated.* ***(B)*** *Growth curve of cells treated with PH and AU at 16°C in the absence of a-factor (average of three independent strains +/- SD).* ***(C)*** *Analysis of mRNA expression changes over time by RT-qPCR upon addition of AU. The gene IMD2 is known to respond to AU and taken along as an indicator of the experimental condition. RNA levels in two Chrom-3xBC libraries are shown relative to an untreated library (Pre) and normalized to a transcript from a spike-in of untreated S. pombe cells (see Materials and Methods).*

Supplemental Figure S3

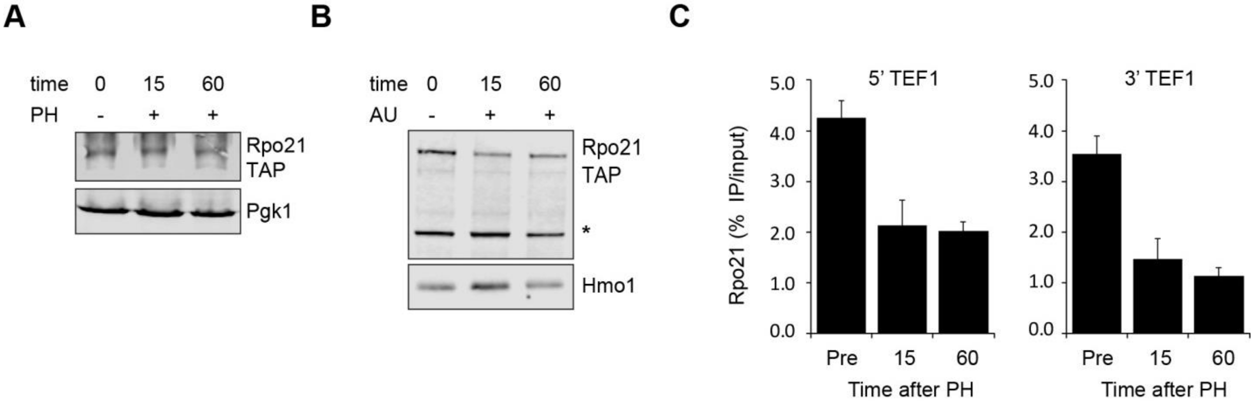

Supplemental Figure S3

(A-B) *Replicates of samples shown in Fig. 3C-D. Immunoblot analysis of Rpo21-TAP with and without PH and AU treatment in G1-arrested cells at 16°C. Pgk1, Hmo1, and a non-specific band (*) were used as loading controls.* ***(C)*** *ChIP-qPCR analysis of Rpo21 binding at the 5' and 3' end of the endogenous TEF1 gene in G1-arrested cells treated with (15 and 60 min) and without (Pre) PH at 16°C (average of three biological replicates +/- SD).*

Supplemental Figure S4

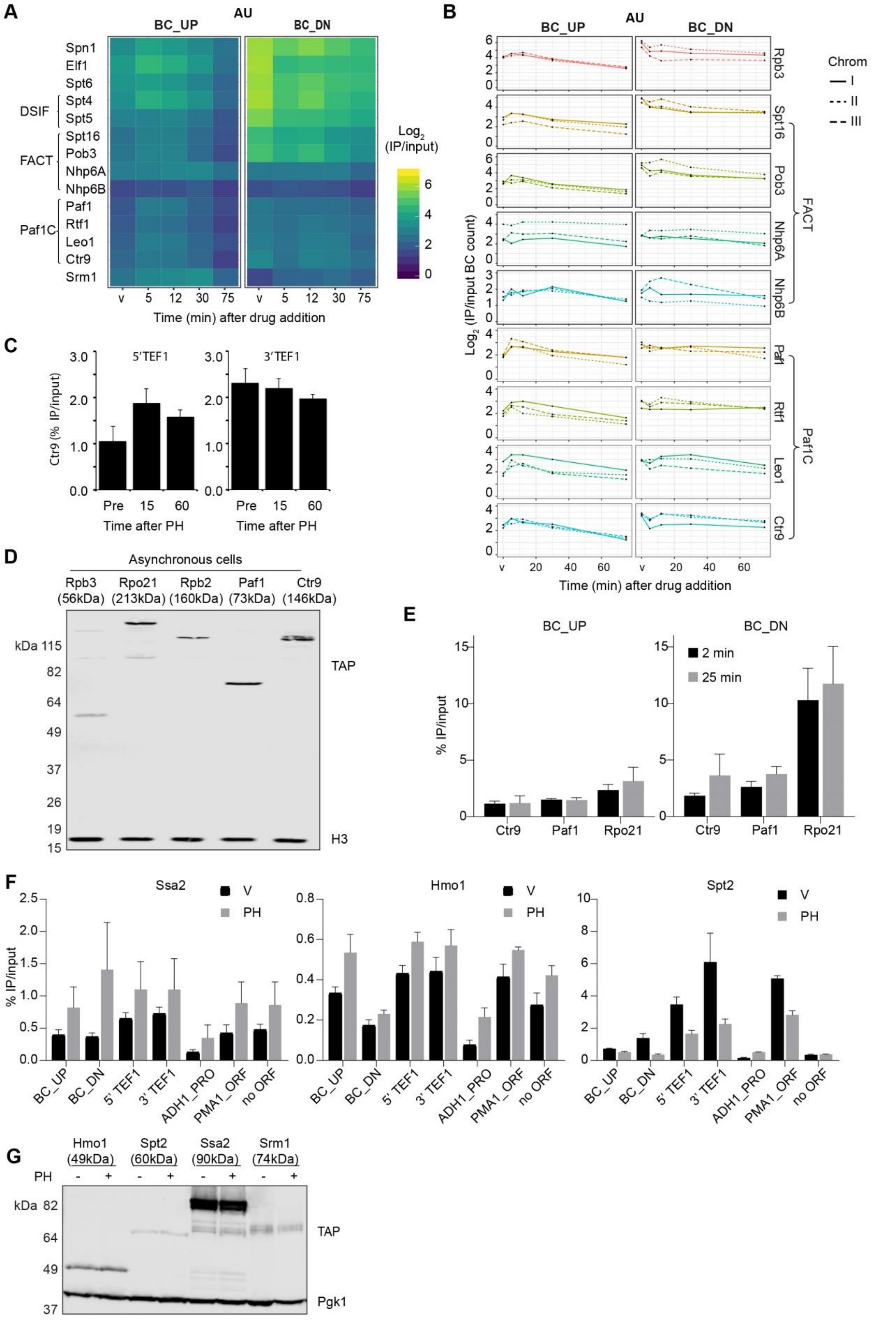

Supplemental Figure S4

(A) *Zoom in on heat map of Fig 2 (AU treatment), showing proteins annotated to transcription elongation.* ***(B)*** *Independent replicates of proteins related to FACT and Paf1C (*Log_2 *IP/input at time points as in Fig. 2; the lines show the three different barcode pairs of the indicated TAP-tagged proteins in the Chrom-3xBC library).* ***(C)*** *ChIP-qPCR analysis of Ctr9-TAP binding at the 5' and 3' end of the endogenous TEF1 gene in G1-arrested cells treated with (15 and 60 min) and without (Pre) PH at 16°C (average of three biological replicates +/- SD).* ***(D)*** *Immunoblot analysis of TAP-tagged Rpb3, Rpo21, Rpb2, Paf1 and Ctr9 in asynchronously growing cells. H3 was used as loading control. The indicated protein sizes (kDa) include the TAP tag (21kDa).* ***(E)*** *ChIP-qPCR analysis of cross-linking efficiency of Ctr9, Paf1, and Rpo21 at the BC_UP and BC_DN regions after 2 and 25 minutes of crosslinking in asynchronous cells grown at 30°C.* ***(F)*** *ChIP-qPCR analysis of binding of Ssa2, Hmo1 and Spt2 in G1-arrested cells at 16°C, treated for 15 min with vehicle (V) or phenanthroline (PH) (average of three biological replicates +/- SD). Analyzed loci are as in Fig. 4E.* ***(G)*** *Immunoblot analysis of TAP-tagged Hmo1, Spt2, Ssa2, and Srm1 in G1-arrested cells treated for 15 minutes with vehicle or PH at 16°C. Pgk1 was used as loading control. The indicated protein sizes (kDa) include the TAP tag (21kDa).*

Supplemental Figure S5

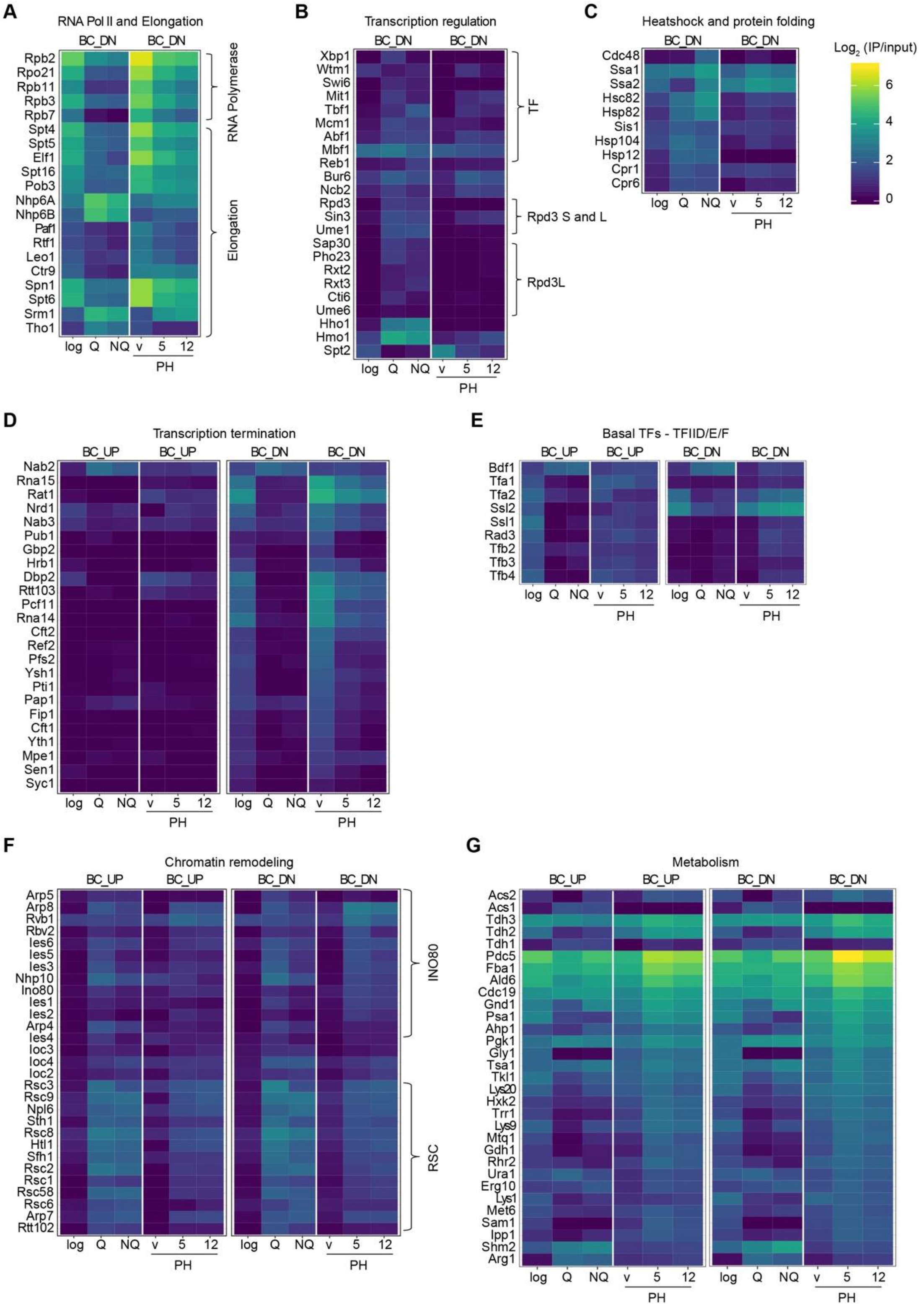

Supplemental Figure S5

(A-G) *Zoom in on heat maps of Fig 5 showing proteins in the indicated annotated clusters. PH treatment during G1 arrest (vehicle, 5 and 12 minutes) is shown for comparison.*

Supplemental Materials and Methods

Yeast strains and library manipulations

Yeast strains used in this study are listed in Supplemental Table S4. Library manipulations on solid media were performed using synthetic genetic array (SGA) technology (Tong and Boone 2006) and a RoToR instrument (Singer Instruments, Watchet, UK). Rearraying of yeast libraries and generation of pools for barcode verification and identification were performed in liquid media using a Hamilton Microlab Star (Hamilton, Germany). The HO locus barcoder library (Korthout et al. 2018) was expanded from ~1100 strains to ~2500 strains using additional barcoder strains kindly provided by the Andrews lab (Douglas et al. 2012). The strains that could be validated were included for Epi-Decoder analysis. The barcoder strains were arrayed over 6 × 384 well plates (Supplemental Fig S1A). The previous and newly acquired barcoder strains were validated by sequencing pools of rows and columns (Korthout et al. 2018) to ensure that the plate location matched with the expected BC_UP and BC_DN sequences. Strains with incorrect or uncertain annotations were removed from further analysis. The updated barcoder library (NKI8591) was combined with a re-arrayed version of the MATα TAP-tag collection (NKI8592) with four key design principles. First, a subset of ~700 chromatin associated factors was allocated to 2 dedicated plates of the 12 × 384 plates (Supplemental Fig S1A, asterisk). The Chromatin-TAP-tag subset contains factors that were either (a) considered a binder at BC_UP or BC_DN, in untreated or HU-treated cells in previous Epi-Decoder experiments at the HO locus (Korthout et al. 2018), or (b) had a GO or GO Slim term related to chromatin, chromatin organization, DNA replication, DNA repair, or transcription (in publicly available datasets from yetfasco (http://yetfasco.ccbr.utoronto.ca), yeastract (http://www.yeastract.com), or in published literature). Factors specifically involved in RNA PolI or RNA PolIII transcription were omitted. The highly-abundant histones were also omitted to increase the sequencing depth of the barcodes associated with other chromatin proteins. Finally, factors that appeared to be incorrectly tagged or barcoded were removed from the analysis. Second, the barcoder library can be re-shuffled to obtain three libraries in which each TAP-tagged protein is coupled with three different barcode pairs, I (NKI8598), II (NKI8599) and III (NKI8600). Third, each full library of ~4000 strains can be processed in two subset pools (Fig 1A, Supplemental Fig S1). Fourth, the three barcode versions of the 700 Chromatin TAP-tag subset (Chrom-3×BC; I (1A and 10D), II (1C and 10F) and III (1B and 10E)) can be processed together in one pool due to non-overlapping barcode pairs. The barcodes and TAP tag were verified for a few selected strains. Strains with incorrect or uncertain barcode or tag status were removed from further analysis.

Culture media and growth conditions, inhibitors

Yeast media were prepared as previously described (van Leeuwen and Gottschling 2002; Tong and Boone 2006; Korthout et al. 2018). For Epi-Decoder analysis of the full libraries, strains were grown in YEPD (1% yeast extract, 2% bacto peptone, and 2% glucose) to log phase at 30˚C and harvested. For screening with transcription inhibitors, the Chrom-3×BC library was grown on YEPD plates overnight and the colonies were pooled in liquid culture. The cultures were grown until log phase (OD660 of ~0.4) at 16˚ C and then a-factor (final concentration of 0.02 µg/ml from a stock solution of 0.2 mg/ml in methanol (O'Reilly et al. 2012) was added for 3 hours to arrest the cells in G1. The arrest was verified by flow cytometry as described below. Synthetic a-factor was synthesized as described (O'Reilly et al. 2012). Samples were collected for Epi-Decoder (150 mL) and RNA isolation (10 mL). 1,10-Phenanthroline (PH; 100 µg/mL final concentration from a 100 mg/mL stock solution in ethanol, Sigma-Aldrich, Cat No. 131377-5G), 6-azauracil (AU) (2 mg/mL final concentration from a 100 mg/mL stock solution in DMSO) or vehicle (ethanol 0.1% or DMSO 2%) was added to G1 arrested cells and samples for Epi-Decoder (150 mL), flow cytometry (1 mL) and RNA extraction (10 mL) were collected at 5 min, 12 min, 30 min and 75 min (for drugs) and 75 min (for vehicle control). All cultures were maintained at 16°C. Drug

concentrations were chosen based on previous studies (Grigull et al. 2004). For validation experiments, RNA extraction and protein extraction, individual strains from fresh plates were grown until log phase (OD660 of ~0.4) at 16°C prior to arresting in G1 and exposure to drug treatment. For growth curves, PH and AU were added to asynchronous cultures of three independent strains (NKI8528, NKI8529, NKI8613) grown in YEPD at 16°C, and samples for OD measurement at 660 nm were collected every 30 min. For isolation of quiescent cells, frozen aliquots of the pooled Chrom-3×BC library were thawed, expanded in YEPD and further processed as described previously (Spain et al. 2018).

RNA isolation and reverse transcription

RNA expression was determined as described previously (Korthout et al. 2018). Briefly, RNA was isolated using the RNeasy Mini Kit (QIAGEN) using the protocol for yeast cells, with a few modifications, essentially as in (Korthout et al. 2018) and with a spike-in reference. Samples of 10 mL were collected at each time point for RNA extraction. A reference *S. pombe* strain (A8545; a gift from R. Allshire) was grown to OD660 = 0.5 and pellets of aliquots were frozen. The equivalent of 5 mL reference culture was added to each 10 mL sample prior to cell lysis. The combined cells were spun down and pellets were resuspended in 600 µL cold RLT buffer. Cells were broken by bead beating with Zirconia/silica beads and debris was removed by centrifugation. The supernatant was collected and mixed with one volume 70% EtOH and transferred to RNeasy columns. Following the buffer RW1 and buffer RPE wash steps RNA was eluted in 50 µL elution buffer. Eluted RNA was treated with DNase I (QIAGEN) to remove genomic DNA. Next, cDNA was prepared using SuperScript II reverse transcriptase (Invitrogen). RT-PCR was performed with the primers described in (Supplemental Table S5). Each sample was measured in two technical duplicates and the average value was taken when combining biological replicates. The *S. pombe* gene *ACT1* was used for normalization. To confirm effects of drug treatment, RNA was isolated from Chrom I, Chrom II and Chrom III libraries and the clonal line NKI8619.

Epi-Decoder (TAG-ChIP-Barcode-Seq)

Epi-Decoder was done essentially as described previously (Korthout et al. 2018). Following arrest and treatment with drugs, samples (150 mL) were immediately crosslinked with 1/10th of the volume of freshly prepared Fix Solution (1% methanol free formaldehyde (Thermo Fisher Scientific; 28908), 50 mM Hepes-KOH, pH 7.5, 100 mM NaCl, 1 mM EDTA) for 20 minutes (unless specified otherwise) and subsequently quenched for 5 minutes with Glycine (125 mM final concentration). Cells were washed once in cold TBS with 0.2 mM PMSF and the pellet was frozen at −80°C. Cells from frozen pellets were lysed by bead beating in 200 µL breaking buffer (100 mM Tris pH 7.9, 20% glycerol, protease inhibitor cocktail EDTA-free) with Zirconia/silica beads and lysates were collected after washing twice in 1 ml FA buffer (50 mM HEPES-KOH pH 7.5, 140 mM NaCl, 1 mM EDTA, 1% Triton X-100, 0.1% Sodium-deoxycholate, protease inhibitor cocktail EDTA-free). Lysates were sonicated using the Bioruptor PICO (Diagenode) for 10 minutes at 30 second intervals and soluble chromatin was separated by centrifugation. For ChIP , 50 µL chromatin was used as input material and 1 mL was used for IP using IgG Sepharose 6 Fast Flow beads (GE healthcare). Beads were washed 3 times with PBSB (PBS containing 5 mg/mL BSA), and incubated with chromatin overnight on a turning wheel at 4˚C. Samples were washed twice in FA buffer, twice in high salt FA buffer (500 mM NaCl), twice in RIPA buffer (10 mM Tris pH 8, 250 mM LiCl, 0.5% NP-40, 0.5% sodium-deoxycholate, 1 mM EDTA) and once with TE buffer (10 mM Tris pH8, 1 mM EDTA). For washing, the Sepharose beads were spun for 2 minutes at 3000 rpm at 4˚C. DNA was eluted from IP samples using 100 µL elution buffer (50 mM Tris pH 8, 10 mM EDTA, 1% SDS), at 65°C, for 10 minutes. IP and input samples were treated with 0.5 µL RNase A (10 mg/mL) and 10 µL ProtK (10 mg/mL) in 70 µL TE for 1 hour at 50°C and subsequently kept overnight at 65°C to reverse crosslinks. DNA was purified using the QIAquick PCR purification kit (Qiagen). BC_UP, BC_DN were amplified separately with specific primers (Supplemental Table S5). PCR products were mixed in an

equimolar fashion and purified from an agarose gel with a size selection of 100-150 bp. The purified DNA was sequenced (single read, >50 bp) on a HiSeq2500/MiSeq platform (Illumina, San Diego, CA), using one or a mix of custom sequencing primers (Supplemental Table S5).

Barcode counting

The barcode counts were obtained as in (Korthout et al. 2018). Briefly, the Perl script eXtracting Counting and LInking to Barcode References (XCALIBR, https://github.com/NKI-GCF/xcalibr) was used to locate the barcodes in the reads and generate a count table. The median of each sequence library (every tag and time-point combination is a separate library) was used to normalize the raw barcode counts. Log_2 IP/input values were used for heatmaps and line plots.

ChIP-qPCR

ChIP experiments on individual clones (NKI8587, NKI8619, NKI8625, NKI2608, NKI2609, NKI2611, NKI2612) were performed similar to Tag-ChIP-Barcode-Seq, but with 20 µL bed volume of IgG Sepharose 6 Fast Flow beads or 40 µL epoxy-activated Dynabeads coupled with rabbit IgG and 200 or 400 µL chromatin (as described previously (Korthout et al. 2018; Vlaming et al. 2019)). Each ChIP was done in triplicate. Quantitative PCR was performed on the purified DNA with SensiFAST SYBR master mix (Bioline) according to the manufacturer's protocol and analyzed on LightCycler 480 II (Roche). The binding was analyzed with primers specific for regions in proximity to the BC_UP and BC_DN or for endogenous regions (Supplemental Table S5). Each sample was measured in two technical duplicates and the average value of these two was taken as one value when combining biological replicates.

Flow Cytometry

Flow cytometry samples were prepared to verify G1 arrest by a-factor and to monitor cell cycle progression in the presence of drugs. Approximately 1×10^7 cells were collected and fixed with 70% ethanol and stored at -20 °C. Flow cytometry was performed as previously described (Haase 2004), after staining DNA with Sytox green (Molecular Probes). Flow cytometry measurements were taken on a FACSCalibur with CellQuest software (Becton Dickinson) and further analyzed with FlowJo software (Treestar).

Protein detection by immunoblot and antibodies

For immunoblotting, strains (NKI8587, NKI8619, NKI8625, NKI8628) were grown to mid-log phase (OD660 0.6–0.9). Samples of 2×10^8 cells were harvested and washed with Tris-EDTA (TE; 10 mM Tris pH 8, 1 mM ethylenediaminetetraacetic acid (EDTA)) containing 0.2 mM phenylmethane sulfonyl fluoride (PMSF). Cell pellets were stored at −80°C until further processing, but at least 30 min. Whole-cell extracts were prepared in SUMEB (1% sodium dodecyl sulfate (SDS); 8 M urea; 10 mM 3-(N-morpholino)propanesulfonic acid, pH 6.8; 10 mM EDTA; 0.01% bromophenol blue) containing protease inhibitors (1 mM PMSF, 1 mM dithiothreitol, 5 mM benzamidine, 1 µg/ml pepstatin, 1 µg/ml leupeptin) by bead beating. The resulting lysate was incubated for 10 min at 65°C and subsequently clarified by centrifuging 5 min at 21 × g. Prior to immunoblotting, 4–10 µl of lysate ($\sim 2 \times 10^6$ cells) was separated on a polyacrylamide gel (16% for histones, 8-10% for other proteins, or gradient gels). Separated proteins were transferred to a 0.45-µm nitrocellulose membrane for 1 h (histones) or 2 h (other proteins) at 1 A. Membranes were blocked with phosphate-buffered saline (PBS) containing 2% or 5% Nutrilon (Nutricia) for 1 h, and first antibody incubations (dilutions see below) were performed overnight at 4°C in 4 ml Tris-buffered saline containing 0.05% Tween-20 (TBST) with 2% Nutrilon. After washing three times in TBST, secondary antibody incubation was performed in TBST with 2% Nutrilon and LI-COR Odyssey IRDye 800CW antibody at 1:10 000 for 45 min at room temperature in the dark followed by 10 min washes twice in TBST and once in PBS. Membranes were scanned using an LI-COR Odyssey IR Imager

(Biosciences) and analyzed using Image Studio 2.0 (LI-COR). For density scans, the signal was the sum of the individual pixel intensity values for a shape minus the product of the median intensity values of the pixels in the background (with a border width top/bottom of 3) and the total number of pixels enclosed by the shape (Area): Signal = Sum − (Background × Area). Primary antibodies and their dilutions used in this study are Pgk1 (459250, Invitrogen, RRID:AB_221541; 1:4000), Histone H2B (39238, Active Motif, RRID:AB_2631110; 1:2000), Histone H3 (RRID:AB_2631108 (43); 1:2000), TAP (CAB1001, Thermo Fisher Scientific, RRID:AB_10709700; 1:1000), Hmo1 (Abcam; ab71834, 1:1000). Secondary antibodies used are IRDye 800CW goat anti-Mouse IgG (0.5 mg) 926-32210 Li-COR (RRID:AB_621842) and IRDye 800CW goat anti-Rabbit igg (0.5 mg) 926-32211 Li-COR (RRID:AB_621843).

Supplemental References

Douglas AC, Smith AM, Sharifpoor S, Yan Z, Durbic T, Heisler LE, Lee AY, Ryan O, Gottert H, Surendra A et al. 2012. Functional analysis with a barcoder yeast gene overexpression system. *"G3: Genes, Genomes, Genetics"* **2**(10): 1279-1289.

Grigull J, Mnaimneh S, Pootoolal J, Robinson MD, Hughes TR. 2004. Genome-wide analysis of mRNA stability using transcription inhibitors and microarrays reveals posttranscriptional control of ribosome biogenesis factors. *Mol Cell Biol* **24**(12): 5534-5547.

Haase SB. 2004. Cell cycle analysis of budding yeast using SYTOX Green. *Curr Protoc Cytom* **Chapter 7**: Unit 7 23.

Korthout T, Poramba-Liyanage DW, van Kruijsbergen I, Verzijlbergen KF, van Gemert FPA, van Welsem T, van Leeuwen F. 2018. Decoding the chromatin proteome of a single genomic locus by DNA sequencing. *PLoS Biol* **16**(7): e2005542.

O'Reilly N, Charbin A, Lopez-Serra L, Uhlmann F. 2012. Facile synthesis of budding yeast a-factor and its use to synchronize cells of alpha mating type. *Yeast* **29**(6): 233-240.

Spain MM, Swygert SG, Tsukiyama T. 2018. Preparation and Analysis of Saccharomyces cerevisiae Quiescent Cells. In *Methods in molecular biology (Clifton, NJ)*, Vol 1686, pp. 125-135.

Tong AH, Boone C. 2006. Synthetic genetic array analysis in Saccharomyces cerevisiae. *Methods Mol Biol* **313**: 171-192.

van Leeuwen F, Gottschling DE. 2002. Assays for gene silencing in yeast. *Methods Enzymol* **350**: 165-186.

Vlaming H, McLean C, Korthout T, Alemdehy MF, Hendriks S, Lancini C, Palit S, Klarenbeek S, Kwesi-Maliepaard EM, Molenaar TM et al. 2019. Conserved crosstalk between histone deacetylation and H3K79 methylation generates DOT1L-dose dependency in HDAC1-deficient thymic lymphoma. *EMBO J* **38**(14): e101564.
